# Annotations

**Published:** 1909-05-29

**Authors:** 


### ANNOTATIONS.

### The Late Mr. Meredith.

With the death of Mr. Meredith there has departed a great writer and thinker for whom medical men have distinct cause to feel regard. As has been widely reported, one of the last acts of his life was to send support to a hospital for incurables, coupled with wise words of commendation. But thoughtful members of the medical profession have long owed a debt of gratitude to the author of *Beauchamp's Career*. In drawing the character of Beauchamp's counsellor he has shown, as Shelley began to do in *Prince Athanase*, the outlook on life and affairs—joined to high capacity—which seems to be produced by the combination of temperament for, and practice of, the scientific profession of medicine, or at any rate is often found along with it. This study bulks large in the book; and although an artist of Mr. Meredith's calibre creates, and does not copy, yet quite possibly the picture of the physician of philosophical and political wisdom may owe something to the personality of the late Dr. Bridges. It is all the more impressive because the opposed (in fact, bitterly and violently opposed) type of mind is also represented. Unfortunately, for every reader of *Beauchamp's Career* there are a hundred in the audience of fashionable, ephemeral authors whose chief use of the medical man as a serious character of fiction has been to mark petty class distinctions and gratify reactionary prejudices, although it is to be remarked that this propensity grows much less noticeable in their later works. Whether it is the moderation of advancing years, or the permeation of Meredithian influence, or the strong forward march of medicine, which has caused this little literary habit of sneering at the doctor to be dropped, is not our province to inquire.

### Medical Defence.

We drew attention last year (The Hospital, June 27, 1908, p. 335) to a new principle in that form of insurance which is afforded by medical defence societies, whereby members of the profession can acquire indemnity not only for their own costs in actions brought against them in their professional capacity, but also for damages assessed against them and taxed costs of the other side in connection therewith. At that time the junior of the English defence societies—the London and Counties Medical Protection Society—was about to adopt this form of additional insurance, and ultimately did so. The Council of the Medical Defence Union at that time hesitated to follow suit, apparently from an apprehension that against members thus insured the imputation of negligence would be freely levelled in courts of law. It was pointed out, however, in these pages last year that no practitioner in his senses would really allow himself to become slack or negligent simply because he might be protected against financial loss, which is probably the least serious part of the troubles of defending actions brought for these reasons. At any rate, the Defence Union has now abandoned the objections entertained when the London and Counties led the way, and has now concluded arrangements with a well-known insurance company by which members whose subscriptions are paid can be indemnified against adverse verdicts and costs: an annual premium of 7s. 6d. covers damages up to £2,000, and one of 9s. protects up to £2,500. A number of stipulations about the conduct of actions and appeals are added, which seem perfectly reasonable. The extent to which the profession takes advantage of it, and the results of its working, will, no doubt, be followed with keen interest by the medical world.

### The Medical Profession in India.

It has always been one of the most attractive features of the Indian Medical Service, into which so many of the flower of our young and newly qualified medical men are admitted, that after a period of some five years in regimental employ (that is as medical officer in charge of troops), a large number of important and well-paid civilian appointments are open to the members of the Service. Furthermore, even those who elect to remain on the strictly military side of the establishment are allowed to supplement their official salaries by private practice, provided this does not interfere with any of their regular duties. It appears, however, from a Parliamentary paper (Cd. 4666) published this week, that Lord Morley has come to the conclusion that no further extension of the civil side of the Indian Medical Service can be allowed. A strong effort must be made to reduce the latter by gradually extending th employment of civil medical practitioners recruited in India: that is, in other words, by native Indians. The Government of India point out that one of the prime essentials of their medical service is that it must always be strong enough to meet the requirements of the Indian Army; and it must, therefore, obviously contain more officers than are sufficient to discharge the ordinary work of that army during peace. By throwing open a large number of civilian posts to their military medical officers, the Indian Government not only secures for itself such a reserve, but insures also that it is composed of officers highly efficient in a professional respect. Of recent years the two disturbing factors which Lord Morley seems to have taken into account are the extension of the Indian medical schools, in which rapidly increasing numbers of native Indians are being qualified, and the desire excited among educated Indians to participate in the Crown Services of their country more than they have hitherto been allowed to do. From the medical point of view the teaching of medicine in India is a step that must be applauded with enthusiasm; it will be generations before sufficient practitioners can be provided adequately to provide for the needs of the population. From a sociological view it is to be remarked that quite a fair proportion of those who nowadays gain entrance to the Indian Medical Service by competitive examination are native Indians; and that it would be a great pity to relax a system which has produced such a splendid medical service as that which the Indian Army now enjoys. There seems to be some conflict of opinion between Lord Morley and the Indian Government precisely how far the process of supersession of Europeans by natives is to go; at least, there are marked discrepancies between the ideal laid down in the despatches between these two authorities which are published in the Parliamentary paper in question.

